# The cost-effectiveness of the RSI QuickScan intervention programme for computer workers: Results of an economic evaluation alongside a randomised controlled trial

**DOI:** 10.1186/1471-2474-11-259

**Published:** 2010-11-11

**Authors:** Erwin M Speklé, Judith Heinrich, Marco JM Hoozemans, Birgitte M Blatter, Allard J van der Beek, Jaap H van Dieën, Maurits W van Tulder

**Affiliations:** 1 Research institute MOVE, Faculty of Human Movement Sciences, VU University Amsterdam, Van der Boechorststraat 9, 1081 BT Amsterdam, The Netherlands; 2 Body@Work, Research Centre on Physical Activity, Work and Health, EMGO Institute, VU University Medical Center, Van der Boechorststraat 7, 1081 BT Amsterdam, The Netherlands; 3 Arbo Unie OHS, Daltonlaan 500, 3584 BK Utrecht, The Netherlands; 4 Department of Public and Occupational Health, EMGO Institute, VU University Medical Center, Van der Boechorststraat 7, 1081 BT Amsterdam, The Netherlands; 5 TNO Quality of Life, Polarisavenue 151, 2132 JJ Hoofddorp, The Netherlands; 6 Institute of Health Sciences, Faculty of Earth and Life Sciences, VU University Amsterdam, Amsterdam, The Netherlands, and EMGO Institute, VU University Medical Center, Amsterdam, The Netherlands

## Abstract

**Background:**

The costs of arm, shoulder and neck symptoms are high. In order to decrease these costs employers implement interventions aimed at reducing these symptoms. One frequently used intervention is the RSI QuickScan intervention programme. It establishes a risk profile of the target population and subsequently advises interventions following a decision tree based on that risk profile. The purpose of this study was to perform an economic evaluation, from both the societal and companies' perspective, of the RSI QuickScan intervention programme for computer workers. In this study, effectiveness was defined at three levels: exposure to risk factors, prevalence of arm, shoulder and neck symptoms, and days of sick leave.

**Methods:**

The economic evaluation was conducted alongside a randomised controlled trial (RCT). Participating computer workers from 7 companies (N = 638) were assigned to either the intervention group (N = 320) or the usual care group (N = 318) by means of cluster randomisation (N = 50). The intervention consisted of a tailor-made programme, based on a previously established risk profile. At baseline, 6 and 12 month follow-up, the participants completed the RSI QuickScan questionnaire. Analyses to estimate the effect of the intervention were done according to the intention-to-treat principle. To compare costs between groups, confidence intervals for cost differences were computed by bias-corrected and accelerated bootstrapping.

**Results:**

The mean intervention costs, paid by the employer, were 59 euro per participant in the intervention and 28 euro in the usual care group. Mean total health care and non-health care costs per participant were 108 euro in both groups. As to the cost-effectiveness, improvement in received information on healthy computer use as well as in their work posture and movement was observed at higher costs. With regard to the other risk factors, symptoms and sick leave, only small and non-significant effects were found.

**Conclusions:**

In this study, the RSI QuickScan intervention programme did not prove to be cost-effective from the both the societal and companies' perspective and, therefore, this study does not provide a financial reason for implementing this intervention. However, with a relatively small investment, the programme did increase the number of workers who received information on healthy computer use and improved their work posture and movement.

**Trial registration:**

Trial registration number: NTR1117

## Background

The costs of musculoskeletal disorders are high, with conservative estimates of the economic burden imposed to the U.S. economy, as measured by compensation costs, lost wages, and lost productivity, between 45 and 54 billion US dollar annually, equalling approximately 0.8% of the gross domestic product [1]. Available cost estimates of musculoskeletal disorders from 15 European countries put the cost between 0.5% and 2% of their gross domestic products[2]. These costs include lost production, staff sickness, compensation and insurance costs, losing experienced staff and recruiting and training new staff, and the effect of discomfort or ill health on the quality of work [2].

Work-related arm, shoulder and neck symptoms are common in Europe, with 25% of the workers reporting work-related neck/shoulder pain, and 15% reporting work-related arm pain[2]. Amongst computer workers, the prevalence of neck, shoulder and arm symptoms is high and cross-sectional studies have reported prevalence rates between 10 and 62% [3]. The total yearly costs of arm, shoulder and neck symptoms in the Netherlands due to decreased productivity, sick leave, chronic disability for work and medical costs were estimated at 2.1 billion Euros [4].

To reduce these costs employers implement interventions aimed at reducing these symptoms. One frequently used intervention, which has recently been developed by an occupational health service in the Netherlands, is the RSI QuickScan intervention programme for computer workers. This multidimensional intervention program addresses a broad spectrum of potential risk factors. It consists of a questionnaire that generates a specific risk profile of the target population, followed by a decision tree for selecting tailor-made interventions [5,6]. The key cost of the this program are the costs of purchasing the questionnaire and costs of implementing interventions, such as an information or training session, a visit to the occupational physician, an eyesight test, an individual workplace assessment or a task analysis.

Even though interventions aimed at reducing arm, shoulder and neck symptoms are often used, there is a shortage of high quality studies evaluating the cost-effectiveness of these interventions [7]. Two recent reviews evaluating the effectiveness of preventive interventions did not find strong evidence for the effectiveness of interventions and were, therefore, hesitant to give policy recommendations [8,9]. A recent systematic review by Brewer et al [8] observed a mixed level of evidence for the general question "Do office interventions among computer users have an effect on musculoskeletal or visual health?". Moderate evidence was observed for: (1) no effect of workstation adjustment, (2) no effect of rest breaks and exercise, and (3) a positive effect of alternative pointing devices. A systematic review by Boocock et al [9], on interventions for the prevention and management of neck/upper extremity musculoskeletal conditions, found moderate evidence for changes to workstation equipment and some evidence that multiple modifier interventions including or excluding exercise can have positive effects in computer workers with arm, shoulder and neck symptoms.

Because resources to achieve the desired positive effects are often scarce, employers and policy makers need to choose the most cost-effective intervention. This has caused a rapid expansion of research on the economics of occupational health in recent years [10]. To be able to make evidence-based choices on which interventions to implement, reliable information is required on both costs and benefits of interventions. Economic evaluations aim to provide this information. The objective of this study was to evaluate the cost-effectiveness and cost-benefits, from both the societal and companies' perspective of the RSI QuickScan intervention programme for computer workers.

## Methods

### Design and study population

This economic evaluation was conducted alongside a cluster Randomised Controlled Trial (RCT). Measurements took place at baseline, after 6-months and 12-months. Cost-effectiveness was determined after 12 months. Although the underlying mechanisms for neck, shoulder and arm symptoms are still poorly understood [11], intervention studies [12,13] and clinical trials [14,15] that proved to be effective suggest that interventions might be effective on short term, i.e. within 6 months.

The study population consisted of computer workers from 7 Dutch organizations in various branches, in different regions of the Netherlands. Workers with and without arm, shoulder and neck symptoms were included. Of the 1,673 persons who were invited to participate in the study, 1,183 persons (71%) completed the baseline questionnaire. A total of 638 persons (54%) participated at baseline as well as at 6- and 12-months follow-up and were included in the statistical analyses.

The study design, protocols, procedures and informed consent form were approved by the Ethics Committee of the Faculty of Human Movement Sciences of the VU University Amsterdam, and all participants electronically provided informed consent before filling out the baseline questionnaire.

The methodological details of the trial are reported in full elsewhere [6].

### Randomisation

The participants were assigned to either the intervention group or the usual care group by means of cluster randomisation (N = 50). To prevent unbalanced randomisation, workers were pre-stratified by the Human Resource Management (HRM) departments of the participating organizations. Organizations were asked to form clusters of approximately the same size and with a comparable amount of computer work. Teams or departments were left intact to avoid crossover effects and to enhance compliance within the intervention groups. The clusters from each organisation were randomly divided into an intervention group and a usual care group. Participants were not informed about their allocation.

### Data collection

At baseline, 6- and 12-months follow-up, the workers completed the internet-based RSI QuickScan questionnaire [5] on exposure to risk factors, and the 7-days and 6-months prevalence of arm, shoulder and neck symptoms. The RSI QuickScan investigates the presence or absence of potential risk factors, such as work posture and movement, job decision latitude, relation with management and colleagues, work pace and load, work environment factors, and furniture. A detailed description of the questionnaire can be found at additional file 1: http://www.rsiquickscan.com/research/questionnaire.pdf. For this study, supplementary questions on the use of medical, alternative care resources and the use of pain medication were added.

### Intervention group

The intervention group received full feedback on their RSI QuickScan questionnaire results. This feedback consisted of scores on a scale from 1 to 10, an interpretation of the score and elaborate advice on the specific actions that they could personally take in order to reduce exposure to risk factors. If workers reported severe symptoms in the arm, shoulder and neck region, their occupational physician invited them for a consultation. Furthermore, from the information given by the respondents, a risk profile was made, using a traffic light risk assessment system. The risk profile was compiled also for each cluster. If more than 30% of the participants in a cluster had a red score or more than 60% of the participants in a cluster had a red or amber score on a certain risk factor, a tailor-made intervention programme was proposed. Per risk factor a (set of) intervention(s) to be advised to the participating organizations was pre-defined.

A set of 16 interventions aimed at reducing the risk factors in the RSI QuickScan was available. Examples of proposed interventions were: at the individual level: an individual workstation check and an eyesight check; at the group level: an education programme on the prevention of arm, shoulder and neck symptoms and training on handling stress in the workplace. A description of all interventions can be found at additional file 2: http://www.rsiquickscan.com/research/interventions.pdf.

### Usual care group

The usual care group did not receive elaborate advice on the specific actions that they could personally take after completing the RSI QuickScan, but more general and limited advice. Furthermore, they did not receive interventions based on the risk profile during the time of the study. Given ethical considerations, workers who reported severe symptoms in the arm, shoulder and neck region, 35 cases in this group, were invited by their occupational physician for a consultation. For other supplementary actions the usual care group was put on a waiting list. Consequently, the usual care group received interventions that were similar to those in the intervention group, but only after the study had ended.

### Outcome measures

For the economic evaluation, the outcome measures were the same as those in the study evaluating the effectiveness of the RSI QuickScan[6], namely, exposure to risk factors [5], the prevalence of arm, shoulder and neck symptoms, and the number of days of sick leave. The prevalence of arm, shoulder and neck symptoms was estimated with the questionnaire, which specified 7 areas in the arm, shoulder and neck region. The total symptom score was a continuous measure that consisted of the sum of points scored on the scale arm, shoulder and neck symptoms.

Sick leave was assessed for the 6-month period prior to baseline, and for 6- and 12-month follow-up. This information was gathered from company records provided by the HRM Department, with the advantage of good coverage, accuracy and consistency [16]. The data consisted of total sick leave, maternity leave excluded, and not solely sick leave due to arm, shoulder and neck symptoms.

### Cost measurement and valuation

Cost-effectiveness analyses were conducted from both the employers' perspective and the societal perspective. The workers use of medical, alternative care resources and the use of pain medication were measured at baseline and at 6- and 12-month follow-up, using an online questionnaire. These data were used to calculate the direct costs of neck, shoulder and arm symptoms. In the online questionnaire, the workers were asked whether they had used pain medication, anti-inflammatory drugs or a combination of both, due to neck, shoulder and arm symptoms, but not what kind or how many. These costs were therefore estimated and results were subjected to a sensitivity analysis. The costs of 40 tablets of pain medication, anti-inflammatory drugs or a combination of both, were imputed for workers who had used these drugs in the past 6-months period. The costs of visits to a general practitioner, medical specialist and physiotherapist were estimated according to the Dutch manual for costing in economic evaluations [17] and were indexed for 2006, the year in which the trial was performed (Table 1). Other intervention costs, such as the costs of the questionnaire, training and visit to the occupational physician, were provided by the Occupational Health Service and their commercial prices were applied. Costs were determined by multiplying the volume reported on each cost by the estimated costs per unit. An overview of these costs per unit can be found in Table 1. The costs of private purchases of specific products/tools aimed at reducing neck, shoulder and arm symptoms were taken into account. This information was derived from the questionnaire by two specific questions about this topic. Since these costs may be underreported and are therefore subject to some uncertainty, the effects of a 500% increase of these costs were estimated in a sensitivity analysis. Indirect costs of productivity loss were also taken into account. These costs are not related to health care, but are costs as a consequence of these symptoms, such as sick leave of productive persons in paid labour. Indirect costs caused by production losses were estimated using the friction cost approach, which assumes that costs are limited to the friction period (i.e. the time it takes to find a replacement), and that the decrease in productivity is less than 100% of the time lost at work (i.e. elasticity) [18]. The friction period was estimated to be 154 calendar days and an elasticity of 0.8 was used [18]. Calculations were based on a mean income of the Dutch working population and indexed for 2006, according to age and gender [17].

**Table 1:** Prices used in the economic evaluation.

	**€ (2006 values)**^**1**^
**Direct health care costs**	
- General practitioner [per visit]^2^	21.03
- Medical specialist [per visit]^2^	102.01
- Physiotherapist and alternative therapist [per visit]^2^	23.68
- Occupational physiotherapist (1 hour)^3^	121.50
- Occupational psychologist (1 hour)^3^	126.50
- Occupational physician (20 min)^3^	70.00
**Direct non-health care costs**	
Purchased products aimed at reducing symptoms (range costs)	0 - 50
**Intervention costs**	
- RSI Quickscan - questionnaire^3^	15.00
- Information session 'Computer work and RSI'^3^	30.00
- Training RSI and Stress^3^	90.00
- Consult occupational physician (20 min)^3^	70.00
- Eyesight test^3^	20.00
- Individual workplace assessment^3^	330.00
- Task analyses^3^	60.00
**Indirect costs**	
Sick leave from paid labour (range costs per hour)^2^	20.89 - 49.78

^1 ^€ = US $ 1.27; ^2 ^prices according to "Standardisation of costs: the Dutch manual for costing in economic evaluations", Oostenbrink, 2004. Indirect costs for paid labour were calculated according to the friction cost approach on the basis of the mean income of the Dutch population stratified for age and gender [17]; ^3 ^prices according to the professional organisation (Occupational Health Service - Arbo Unie)

### Statistical analysis

Only workers who completed all three measurements and questionnaires were included in the analyses. Analyses to estimate the effect of the intervention were done according to the intention-to-treat principle [19]. Resource use, sick leave and costs were calculated per person for the 12-months follow-up period.

Cost data are usually skewed to the right [20]. To compare costs between groups, confidence intervals for cost differences were computed by bias-corrected and accelerated (BCa) bootstrapping with 2000 replications [21]. Non-parametric bootstrapping is often used to analyze cost data, because decision makers need to be able to link the summary measure of cost per person to the overall budget impact and this can only be achieved by the mean [22]. The scores were, therefore, expressed as the mean costs per person for the intervention and usual care group and the difference in mean costs between both groups over 12 months. Costs for paid labour were adjusted for 2006 values.

Cost-effectiveness analysis relates the difference in costs between the intervention and the usual care groups to the difference in effects. A cost-effectiveness analysis was performed on the two risk factors ("information" and "work posture and movement") that showed a significant positive change in the randomized controlled trial assessing the effects of the RSI QuickScan and on two factors that did not show a significant positive change, "arm, neck, shoulder symptoms" and the number of "days of sick leave"[6]. For the cost-effectiveness analysis, effect scores on the scales "information", "work posture and movement" and "arm, neck, shoulder symptoms" were adjusted for baseline. In this analysis, we used the total costs for the outcomes risk factors and prevalence.

In a cost-benefit analysis the effects are expressed as benefits in monetary units. The difference in the monetary costs due to sick leave between the intervention group and the usual care group was calculated. For the cost-effectiveness and cost-benefit analyses, the number of days and costs of sick leave were calculated for the half year period, starting 6-months prior to the last measurement and adjusted for the half year period 6-months prior to baseline. For the cost-effectiveness analysis of the outcome sick leave from a societal perspective, only the direct costs were included to avoid double counting. From the companies' perspective, sick leave is a real expense in the Netherlands where the employer pays 100% of the wage during the first year of sick leave, We included indirect costs of sick leave as benefit in the cost-benefit analysis performed from the companies' perspective.

To estimate the incremental cost-effectiveness ratio (ICER), we divided the incremental costs of the intervention group compared with those of the usual care group by the incremental effects for each of the effect measures separately. The uncertainty associated with the incremental cost-effectiveness ratios was analysed by bootstrapping using the bias-corrected percentile method with 5000 replications [23]. The bootstrapped incremental cost/effect pairs were plotted on a cost-effectiveness plane [24], consisting of four quadrants with a horizontal axis indicating the effectiveness of the intervention in relation to the usual care group and the vertical axis indicating the difference in costs between the groups.

Sensitivity analyses were performed in which missing cost data for medicine use were imputed and the costs of private purchases of specific products/tools was increased.

## Results

### Resource use and costs

In both groups, resource utilization was low and there were no significant differences between the two groups (Table 2). Physical and alternative therapy were the most frequently used health care resources in both groups, with the highest cost. Approximately 14% of the participants in the study purchased products aimed at reducing symptoms, such as a special computer mouse. However, the mean costs of these products were low compared to the other direct costs, especially compared to sick leave (Table 2).

**Table 2:** Utilization, costs and differences in costs during the 12-months follow-up period.

Resource use and costs	Intervention (n = 320) Mean (SD)	Usual care (n = 318) Mean (SD)	Intervention - Usual care Difference in costs Mean (95% CI)
General practitioner			
- no of visits	0.37 (1.43)	0.43 (1.39)	
- costs^2^	7.69 (30.12)	9.12 (29.22)	-1.44 (-6.05; 3.18)
Medical specialist			
- no of visits	0.25 (1.37)	0.28 (1.26)	
- costs^2^	25.50 (140.14)	28.55 (128.38)	-3.05 (-23.95; 17.85)
Physical and alternative therapist			
- no of treatment sessions	1.71 (5.68)	1.65 (4.89)	
- costs^2^	40.55 (134.50)	39.02 (115.81)	1.53 (-17.99; 21.05)
Occupational psychologist			
- no of treatment sessions	0.02 (0.21)	0.00 (0.06)	
- costs^3^	2.37 (26.39)	0.40 (7.09)	1.97 (-1.03; 4.98)
Occupational physiotherapy			
- no of treatment sessions	0.17 (1.61)	0.18 (1.06)	
- costs^3^	23.92 (195.11)	21.78 (128.17)	2.14 (-23.54; 27.82)
Occupational health physician			
- no of visits	0.11 (0.68)	0.12 (0.67)	
- costs^3^	7.66 (47.87)	8.59 (46.89)	-0.93 (-8.30; 6.44)
Purchased products aimed at reducing symptoms			
- % yes	14	11	
- costs	0.16 (2.30)	0.03 (0.56)	0.13 (-0.19; 0.44)
Sick leave from paid labour			
- no of days	10.38 (21.31)	12.50 (25.25)	
- costs^2^	1768.18 (3686.11)	2090.78 (4303.91)	-322.60 (-945.48; 300.28)

Utilization, costs and differences in costs (€, 2006 values ^1^) of health care, non-health care resources and sick leave per person in the intervention and usual care group during the 12-months follow-up period. ^1 ^€ = US $ 1.27; ^2 ^prices according to "Standardisation of costs: the Dutch manual for costing in economic evaluations", Oostenbrink, 2004. Indirect costs for paid labour were calculated according to the friction cost approach on the basis of the mean income of the Dutch working population stratified for age and gender [17]; ^3 ^prices according to the professional organisation (Occupational Health Service - Arbo Unie)

### Intervention use and costs

The utilization rates (%) of interventions during the 12-months follow-up period are given in Table 3. All participants, regardless of group allocation, received the RSI QuickScan. There were significant between-group differences in utilization rates for the "Education on the Prevention of RSI for Employees", the training "RSI and Stress", the eyesight check, and task analysis (Table 3). The total mean costs of the interventions were €58.97 and €28.24 in the intervention and usual care group, respectively (Table 4 and 5).

**Table 3:** Utilization rates of interventions, mean intervention costs^2 ^per person and the difference in mean costs.

Type of utilization	Intervention (n = 320)	Usual care (n = 318)	p-value	Intervention - Usual care Difference in costs Mean (95% CI)
RSI QuickScan^3^				
- utilization rate (% yes)	100	100		
- Mean costs	15.00	15.00	.	0
Occupational health physician				
- utilization rate (% yes)	7.8	6.0	0.36	
- Mean costs (SD)	5.90 (21.00)	4.40 (17.90)		1.50 (-1.53; 4.54)
Education on the Prevention of RSI for Employees				
- utilization rate (% yes)	26.3	0.3	0.00	
- Mean costs (SD)	7.88 (13.22)	0.09 (1.68)		7.81 (6.31; 9.25)
RSI and Stress				
- utilization rate (% yes)	24.1	1.9	0.00	
- Mean costs (SD)	14.34 (32.99)	1.13 (10.04)		13.21 (9.42; 17.01)
Eyesight check				
- utilization rate (% yes)	18.8	6.9	0.00	
- Mean costs (SD)	3.75 (7.82)	1.38 (5.08)		2.37 (1.34; 3.39)
Individual Workstation Check				
- utilization rate (% yes)	2.2	0.9	0.21	
- Mean costs (SD)	11.34 (60.22)	6.23 (44.97)		5.12 (-3.15; 13.84)
Task analyses				
- utilization rate (% yes)	1.3	0.0	0.05	
- Mean costs (SD)	0.75 (6.68)	0.00 (0.00)		0.75 (0.02; 1.49)

Utilization rates (%) of interventions, mean intervention costs^2 ^per person and the difference in mean costs (€, 2006 values^1^) between the intervention and usual care groups during the 12-months follow-up period. ^1 ^€ = US $ 1.27; ^2 ^prices according to the professional organisation (Occupational Health Service - Arbo Unie); ^3 ^Since there is no variation in intervention costs within the groups, SDs and 95% confidence intervals can not be calculated.

**Table 4:** Mean costs for the intervention and usual care group and the difference in mean costs from a societal perspective.

Costs	Intervention (n = 320) Mean (SD)	Usual care (n = 318) Mean (SD)	Intervention - Usual care Difference in costs Mean (95% CI)^2^
Total intervention costs^3^	58.97 (84.74)	28.24 (56.11)	30.73 (18.78; 41.03)
Total non-health and health care costs^4^	107.85 (426.32) _________________+	107.49 (284.68) _________________+	0.36 (-60.77; 53.04) ____________________+
Total direct costs	166.82 (436.96)	135.73 (294.55)	31.08 (-22.02; 80.27)

Mean costs per person for the intervention and usual care group and the difference in mean costs (€, 2006 values^1^) between both groups over 12 months. ^1 ^€ = US $1.27; ^2 ^95% confidence interval obtained by bias-corrected and accelerated bootstrapping with 2000 replications; ^3 ^prices according to the professional organisation (Occupational Health Service - Arbo Unie); 4 prices according to "Standardisation of costs: the Dutch manual for costing in economic evaluations", Oostenbrink, 2004

**Table 5:** Mean costs for the intervention and usual care group and the difference in mean costs from an employer's perspective.

Total direct costs	Intervention (n = 320) Mean (SD)	Usual care (n = 318) Mean (SD)	Intervention - Usual care Difference in costs Mean (95% CI)^2^
Total intervention costs^3^	58.97 (84.74)	28.24 (56.11)	30.73 (18.78; 41.03)

Mean costs per person for the intervention and usual care group and the difference in mean costs (€, 2006 values^1^) between both groups over 12 months. ^1 ^€ = US $1.27; ^2 ^95% confidence interval obtained by bias-corrected and accelerated bootstrapping with 2000 replications; ^3 ^prices according to the professional organisation (Occupational Health Service - Arbo Unie).

In both groups, the main contributor to the total direct costs were the total non-health and health care costs, which were slightly, but not significant, higher in the intervention group (Table 4). There was a significant difference in total intervention costs. However, there was no significant difference in total direct costs, which is the sum of all intervention, non-health and health care costs (Table 4).

### Cost-effectiveness

The mean societal costs and effects per person over 12 months for the scales "information", "work posture and movement" and "arm, neck, shoulder symptoms" show a significant positive effect for the intervention group on received information on healthy computer use and on work posture and movement, with a relatively small difference in total direct costs between the groups (Table 6). The reduction of arm, neck and shoulder symptoms was more prominent in the intervention group, but not significantly so (Table 6). Days and costs of sick leave were higher in the intervention group. However, it is important to note that the effects and benefits were highly non-significant, as can be derived from the 95% confidence interval of the difference in days and costs of sick leave (Table 6).

**Table 6:** Total direct costs, effects and the difference in mean costs from a societal perspective.

	Intervention Mean (SD)	Usual care Mean (SD)	Intervention - Usual care Difference costs and effects Mean (95% CI)^2^
**information**	(N = 320)	(N = 318)	
Total direct costs	166.82 (436.96)	135.73 (294.55)	31.09 (-26.70; 88.88)
Effects (range 0 - 1)	-0.22 (0.51)	-0.10 (0.47)	-0.11 (-0.19; -0.04)
**work posture and movement**	(N = 315)	(N = 317)	
Total direct costs	169.23 (440.00)	136.11 (294.94)	33.12 (-25.32; 91.56)
Effects (range 0 - 11)	-0.96 (1.89)	-0.61 (2.00)	-0.35 (-0.66; -0.05)
**arm, neck, shoulder symptoms**	(N = 312)	(N = 308)	
Total direct costs	170.55 (441.91)	128.11 (276.04)	42.44 (-15.48; 100.36)
Effects (range 0 - 44)	-1.36 (5.49)	-0.77 (5.92)	-0.59 (-1.48; 0.31)
**sick leave from paid labour**	(N = 320)	(N = 318)	
Total direct costs	166.82 (436.96)	135.73 (294.55)	31.09 (-26.70; 88.88)
Effects (days of sick leave)	0.14 (23.71)	-0.30 (23.970)	0.44 (-3.26; 4.14)
Benefits^3 ^(costs of sick leave)	307.71 (3122.17)	227.51 (2847.64)	80.20 (-383.45; 543.86)

Total direct costs and effects per person for the intervention and usual care group and the difference in mean costs (€, 2006 values ^1^) between both groups from a societal perspective. Mean costs and effects for the scales "information", "work posture and movement" and "arm, neck, shoulder symptoms are presented over a 12 months period. A negative effect value for these risk factors represents a reduction in exposure and the desired effect. Mean costs and effects for days- and costs of sick leave are presented over a 6 months period. A positive effect or benefit value for sick leave represents an increase in days or costs of sick leave and an undesired effect. ^1 ^€ = US $1.27; ^2 ^95% confidence interval obtained by bias-corrected and accelerated bootstrapping with 5000 replications; ^3 ^Indirect costs for paid labour were calculated according to the friction cost approach on the basis of the mean income of the Dutch population stratified for age and gender [17].

The cost-effectiveness ratio (ICER) for "information" was estimated at -277.58, indicating that the cost of one point of improvement, which in this case is a negative score since it is a reduction in risk, on a scale ranging from 0 (did receive information on healthy computer use) to 1 (did not receive information on healthy computer use) was estimated at €277.58.

**Figure 1:**
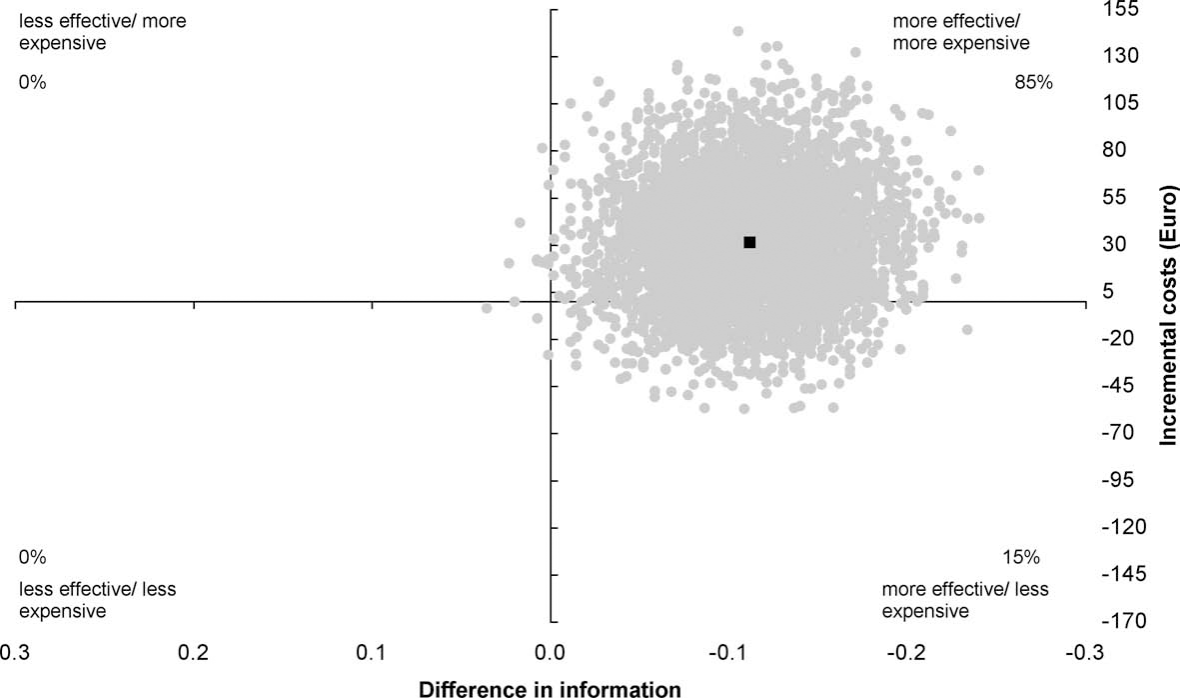
**Cost-effectiveness plane for "Information"**. Intervention versus usual care; range 0 - 1. The individual points on the plane represent 5000 bootstrapped cost-effect pairs using the bias-corrected percentile method. The central black dot indicates the point estimate of the incremental cost-effectiveness ratio.

The intervention is significantly more effective with 85% of the incremental cost/effect pairs located in the northeast quadrant and 15% in the southeast quadrant of the cost-effectiveness plane (Figure 1).

**Figure 2:**
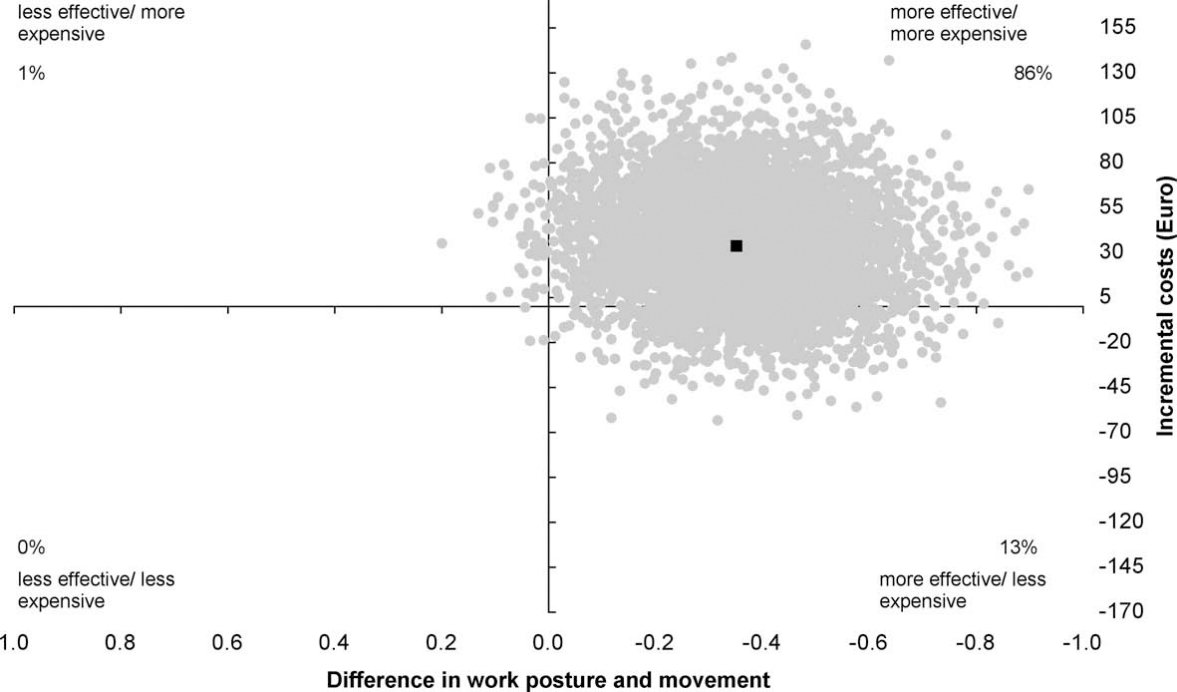
**Cost-effectiveness plane for "Work posture and movement"**. Intervention versus usual care; range 0-11. The individual points on the plane represent 5000 bootstrapped cost-effect pairs using the bias-corrected percentile method. The central black dot indicates the point estimate of the incremental cost-effectiveness ratio.

The cost-effectiveness ratio (ICER) for "work posture and movement" was estimated at -93.82, indicating a cost of €93.82 for one point improvement on a scale ranging from 0 (perfect work posture and movement) to 11 (poor work posture and movement). The intervention was significantly more effective with 86% of the incremental cost/effect pairs located in the northeast quadrant and 13% in the southeast quadrant of the cost-effectiveness plane (Figure 2).

**Figure 3:**
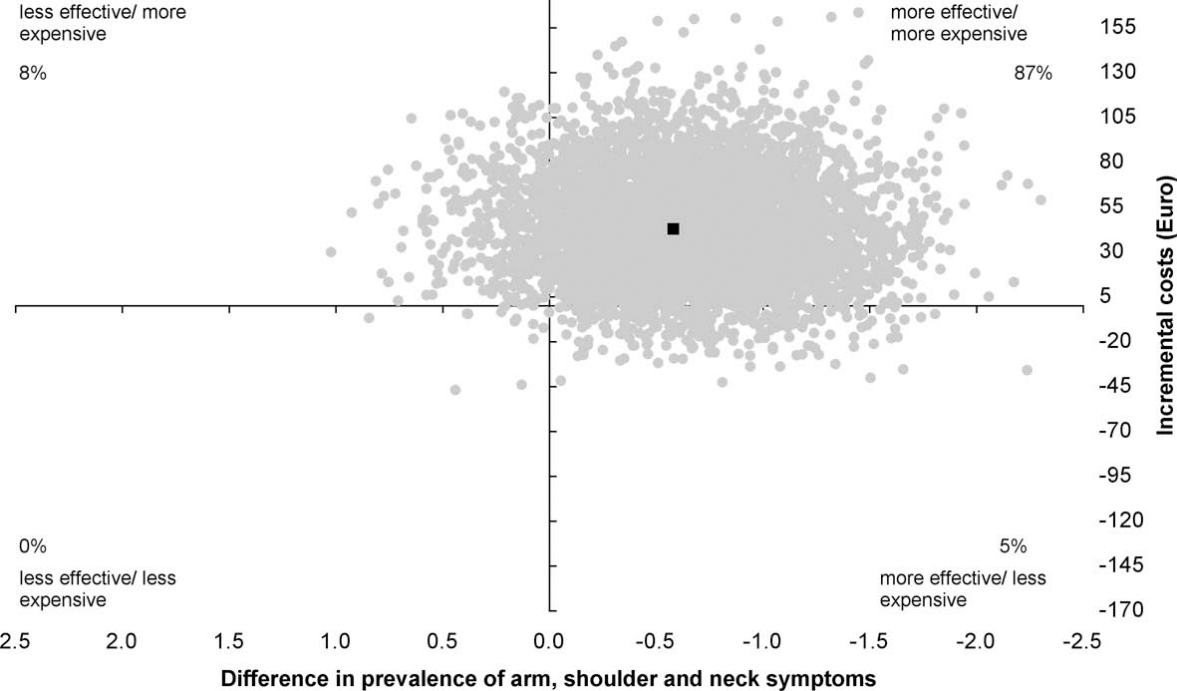
**Cost-effectiveness plane for the prevalence of arm, shoulder and neck symptoms - total symptom score**. Intervention versus usual care; range 0 - 44. The individual points on the plane represent 5000 bootstrapped cost-effect pairs using the bias-corrected percentile method. The central black dot indicates the point estimate of the incremental cost-effectiveness ratio.

The cost-effectiveness ratio (ICER) for "the prevalence of arm, shoulder and neck symptoms" indicated that the cost of one point reduction in arm, shoulder and neck symptoms, on a scale ranging from 0 (no arm, shoulder and neck symptoms) to 44 (severe arm, shoulder and neck symptoms) was estimated at €72.55. The intervention was more effective with 87% of the incremental cost/effect pairs located in the northeast quadrant, 8% in the northwest quadrant, 5% in the southeast quadrant and 0% in the southwest quadrant of the cost-effectiveness plane (Figure 3).

**Figure 4:**
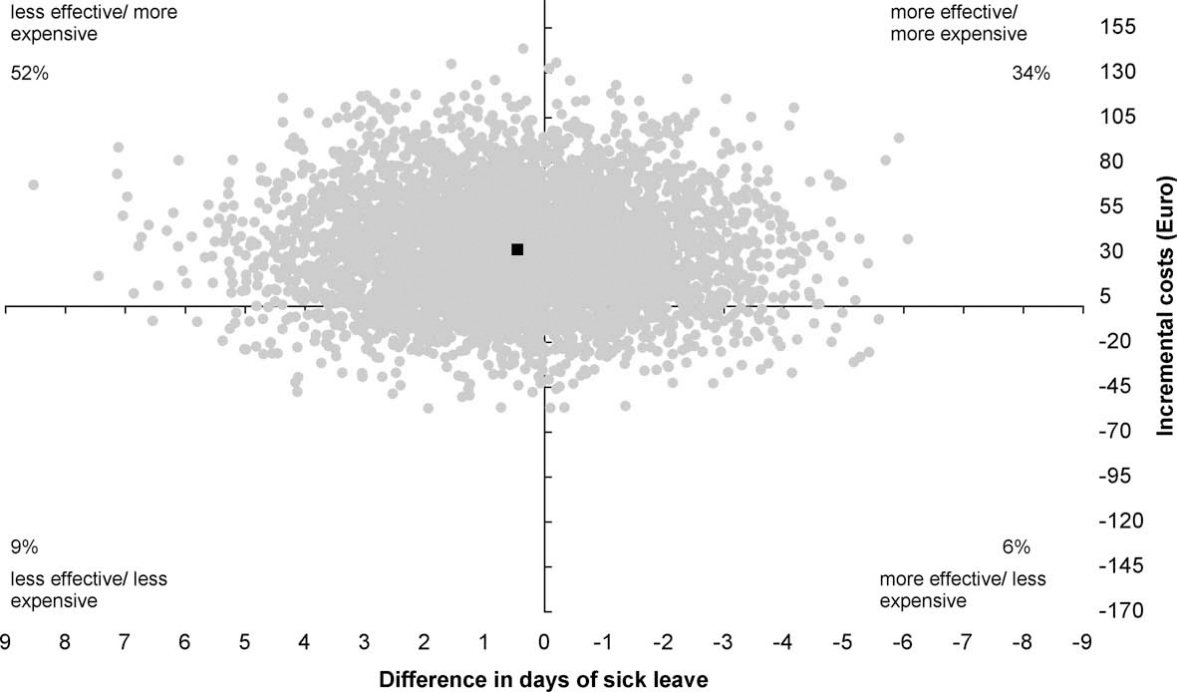
**Cost-effectiveness plane for days of sick leave**. The individual points on the plane represent 5000 bootstrapped cost-effect pairs using the bias-corrected percentile method. The central black dot indicates the point estimate of the incremental cost-effectiveness ratio.

The cost-effectiveness ratio (ICER) for "days of sick leave" indicated that an investment of €71.31 is associated with in an increase of sick leave by one day. Hence, the intervention was generally less effective with 40% of the incremental cost/effect pairs located in the east quadrants, 61% in the west quadrants of the cost-effectiveness plane (Figure 4). This is, of course, an undesired effect. However, it is important to note that the effect is highly non-significant, as can be seen from the costs-effectiveness plane and the 95% confidence interval of the difference in sick leave days, which ranges from -3.26 to 4.14 (Table 6).

### Cost-benefit

**Figure 5:**
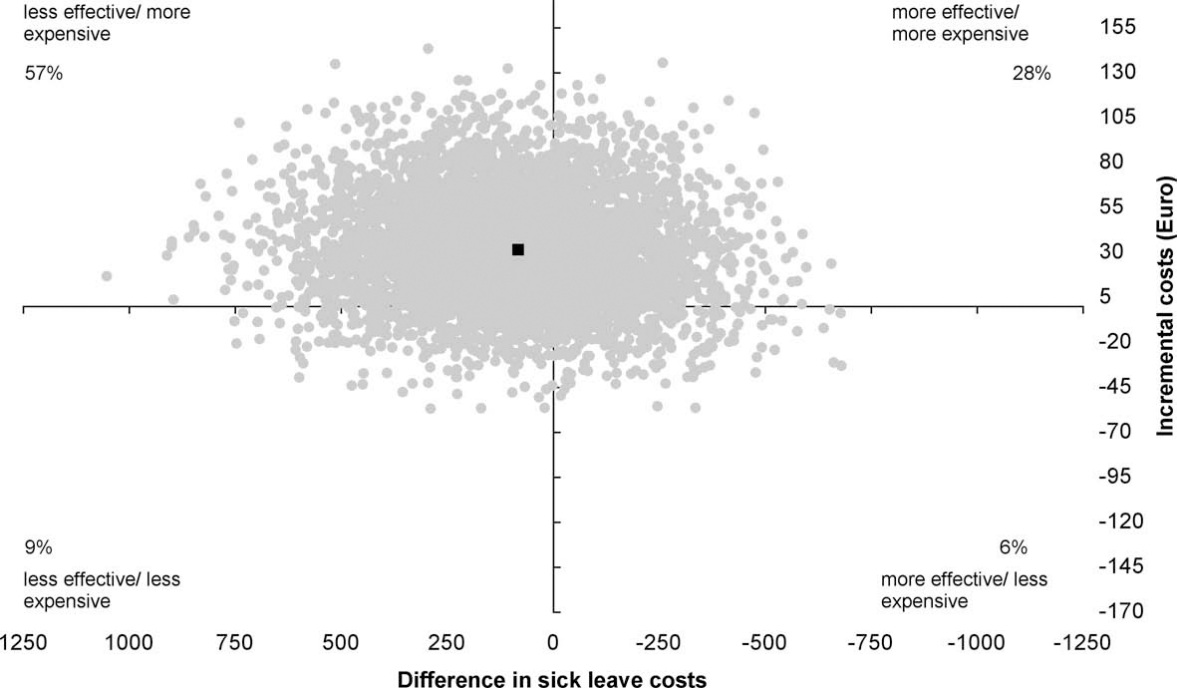
**Cost-effectiveness plane for sick leave costs**. The individual points on the plane represent 5000 bootstrapped cost-effect pairs using the bias-corrected percentile method. The central black dot indicates the point estimate of the incremental cost-effectiveness ratio.

The cost-effectiveness ratio (ICER) per point change in cost of sick leave is estimated at €0.39, which means that an investment of €0.39 is associated with an €1 increase of sick leave costs. Obviously, this is an undesirable, but highly non-significant, effect as can be seen from the cost-effectiveness plane (Figure 5), which had 34% of the incremental cost/effect pairs located in the east quadrants, 66% in the west quadrants and the 95% confidence of the difference in sick leave costs (Table 6).

### Sensitivity analysis

Imputation of missing cost data for medicine use increased the costs in the usual care group by €1.42 (SD = 4.45) and in the intervention group by €1.38 (SD = 4.25). The imputation of missing cost data led to a mean difference of €31.02 (95% CI -32.32 to 76.43) in total costs.

Increasing the costs of private purchases of specific products/tools, aimed at reducing neck, shoulder and arm symptoms, by 500% increased the cost in the usual care group to €0.16 (SD = 2.80) and in the intervention group to €0.78 (SD = 13.98). The imputation of missing cost data led to a mean difference of €31.60 (95% CI -19.57 to 84.30) in total costs.

## Discussion

This study evaluated the cost-effectiveness and cost-benefits of the RSI QuickScan intervention programme on exposure to risk factors, prevalence of arm, shoulder and neck symptoms and sick leave in computer workers. This economic evaluation was performed alongside a cluster randomized trial [6]. The intervention was not cost-effective compared to usual care.

### Resource use and costs

The results show only small and non-significant differences between the two groups in the total health and non-health care resource use and corresponding costs (€0.36) after 12 months follow-up (Table 4). As expected, resource use and costs of the interventions were significantly higher (€30.73) in the intervention group compared to the usual care group. Total direct costs were higher (€31.08) in the intervention group, but the difference was not significant. In this study, indirect costs due to sick leave was an outcome measure. The cost-effectiveness results in this study are primarily viewed from a societal perspective, because this is the broadest perspective where all costs and effects are taken into account, regardless of who benefits from the health effects or who pays for the costs. However, the cost-effectiveness from an employer's perspective, which is highly relevant to decision makers in organizations, can also be derived from the results presented.

The overall conclusion is that results of the cost-effectiveness analysis performed from a societal perspective are similar to the results of the cost-benefit analysis performed from a companies' perspective. This is due to the fact that there was only a small difference in total non-health and health care costs between the intervention and usual care group of 0.36 euro. From a societal perspective the difference in total direct costs between the usual care and intervention group was 31.08 Euro and from the employer's perspective the difference in total direct costs between the usual care and intervention group was 30.73 Euro.

The monetary investments for the interventions that have to be made by the employer are estimated at €58.97 per person. This investment resulted in an increase in sick leave days and sick leave costs and, therefore, this intervention was not cost-effective.

### Limitations of the study

The costs of work-related arm, shoulder and neck symptoms and corresponding sick leave in computer workers are high. One of the major reasons why organizations implement interventions is to decrease these costs [25]. However, the effect of the intervention depends largely on a successful implementation by the management and whether employees are applying the intervention in their daily work or not. In this study, unfortunately, most of the participating organizations did not implement all proposed preventive measures; although they indicated that they would try to do so at recruitment in the study. The advised package of interventions as a whole was accepted by only one of the seven participating organizations. The other organizations chose parts of the proposed intervention plan, while two organizations even decided to do nothing at all. As a consequence, many workers who should have received an intervention were never offered one, let alone participated in one. This may have added to the limited cost-effectiveness of the RSI QuickScan intervention programme. Which factors impeded the implementation of the interventions will be investigated in a process evaluation. However, the main reason given by the organizations was that their available budget for these interventions was insufficient. Further strengths and weaknesses of the cluster randomized trial have been described extensively elsewhere [6].

### Presenteeism

In this study, productivity loss was measured by sick leave. Presenteeism, when the employee is at work, but not fully productive, was not included. Loss of productivity as a result of presenteeism, due to arm, shoulder and neck symptoms, constitutes a substantial economic burden to employers in the Netherlands [4]. The productivity in the case of presenteeism may vary from a relatively small decrease in productivity, to a total loss of productivity. In a study by Martimo et al [26], workers with arm, shoulder and neck symptoms, on average, lost one third of their regular productivity, which in a normal work day would correspond to 2.5 hours of lost working time. In the RSI QuickScan questionnaire the workers were asked to indicate on a scale from 1 to 10 how efficiently they worked on days when they were at work, but were suffering from neck, shoulder and arm symptoms. Unfortunately, the workers were not asked to quantify the duration of this period, which makes it impossible to estimate the associated costs. Consequently, cost of productivity loss in this study may have been underestimated [27].

### Sensitivity analysis

A sensitivity analysis is an important feature in economic evaluations, since study results can be sensitive to the values assumed for by key parameters [22]. The parameters concerning the direct health care costs were estimated according to the prices in the Dutch manual for costing in economic evaluations and adjusted to calendar year 2006 [17]. This manual describes a uniform costing methodology, which makes it easier to interpret and compare studies. The prices in the manual are widely accepted and used for cost-effectiveness studies in the Netherlands. The parameters for intervention costs were the actual prices according to the occupational health service that provided the services. These prices are suitable for use since there is a well functioning, competitive occupational health care market in the Netherlands and the used market prices are not subsidized, nor have they got a high profit margin.

In this study, physical and alternative therapy, such as Cesar/Mensendieck exercise therapy, were the most used health care resources in both groups, with the highest cost. This is quite common in the Netherlands. Approximately 13% of the Dutch population is receiving physical therapy once per year, with an average of 18 visits per person [28].

Imputation of missing cost data for medicine use and increasing the costs of private purchases of specific products/tools aimed at reducing neck, shoulder and arm symptoms in the sensitivity analyses did not change the conclusions of this economic evaluation.

### Comparison with other studies

Studies evaluating the (cost-) effectiveness of interventions aimed at reducing arm, shoulder and neck symptoms are scarce [7-9] and no other cost-effectiveness study, in which an advised set of interventions is based on a previously established risk profile, was found in the literature. Therefore, this article provides new information for decision makers and occupational health professionals on the effectiveness and cost-effectiveness of such an intervention strategy for computer workers. Unfortunately, since no similar cost-effectiveness study was found in the literature, the evidence on the lack of effectiveness of this strategy is limited. Still, a few studies on the cost-effectiveness of preventive interventions aimed at reducing arm, shoulder or neck symptoms, in different working populations, have been published in the last decade. A study by Yeow et al [29] on the cost-effectiveness of simple and inexpensive ergonomic improvements in test workstations of an electronics factory, found average savings in yearly rejection cost (i.e. costs as a results of customers returning defect products), reduction in rejection rate, increase in monthly revenue, improvements in productivity and other benefits. Even though the setting of Yeow's study, an assembly factory in an industrially developing country, was quite different from ours, one of the implemented ergonomic interventions, an optimization of the workstation design, was also one of the interventions used in our study. Several other randomized controlled trials have shown some promising results, but did not include a cost-effectiveness analysis. A study by Ketola et al [30] that evaluated the effect of an intensive ergonomic approach and education on workstation changes and musculoskeletal disorders among computer workers, found that both the intensive ergonomics approach and education in ergonomics did help to reduce discomfort in computer work. A study by Bohr [31] found that computer workers who received education reported less pain/discomfort and psychosocial work stress following the intervention than those who did not receive education.

## Conclusions

In conclusion, the RSI QuickScan intervention programme was not cost-effective compared to usual care. This might be caused by the fact that a large percentage of workers did not receive the advised intervention. For those who did receive an intervention, the duration and intensity of the interventions was often low. However, the programme did increase the number of workers who received information on healthy computer use and improved their work posture and movement at relatively modest costs.

## Competing interests

The first co-author is an employee of Arbo Unie. This non-profit occupational health service is the proprietor of the RSI QuickScan.

## Authors' contributions

ES participated in the design, performed the study, including statistical analysis and drafted the manuscript. MH and JvD participated in the design, helped with the statistical analysis and drafting the manuscript. BB, JH, AvdB and MvT participated in the design of the study, reviewed the paper and made corrections. All authors read and approved the final manuscript.

## Pre-publication history

The pre-publication history for this paper can be accessed here:

http://www.biomedcentral.com/1471-2474/11/259/prepub

## Supplementary Material

Additional file 1 **RSI QuickScan questionnaire**. The file provides a detailed description of the content of the RSI QuickScan questionnaire. Click here for file

Additional file 2 **Interventions in the RSI QuickScan intervention programme**. The file provides a description of all 16 interventions that were part of the RSI QuickScan intervention programme. Click here for file
